# Chemistry of the Oral Secretions and Their Action on the Teeth

**Published:** 1873-12

**Authors:** Edward C. Chase

**Affiliations:** Iowa City.


### AMERICAN JOURNAL

OF

### DENTAL SCIENCE.

Vol. VII. THIRD SERIES--DECEMBER, 1873. No. 8.

### ARTICLE I.

### *Chemistry of the Oral Secretions and Their Action on the Teeth.*

BY EDWARD C. CHASE, IOWA CITY.

(Read before the Illinois State Dental Society.)

Of all the many subjects within the boundless realm of dental science, none can be of more interest and importance to the scientific dentist than the study of the physiological and chemical properties of those fluids which continually bathe the objects of his solicitous care and pecuniary support.

The teeth, constantly washed, as they are, by fluids poured into the mouth from sundry glands, and these fluids impregnated by obnoxious gases resulting from the putrefactive decomposition of particles of food, are absolutely under the influence and control of these liquids. This being the case, then, not only the integrity of the teeth themselves, but the permanence of our operations upon them, depend upon the chemical condition of these oral secretions.

It matters not how thoroughly nature may have impacted the lime salts or closed the enamel seams, if the mixed secre tions contain the fatal acid, whether mineral, amylacious or albuminous, the pearly citadel is besieged and will surely fall. While on the other hand, through ignorance of hygienic laws, or other obscure causes, the vital forces have been arrested or perverted from their legitimate channel, and the teeth consequently but imperfectly calcified, still they will be able to maintain their integrity of form so long as their aqueous surrounding is free from the insiduous enemy, *acid.*

Under the head of oral secretions are included the products of all those glands whose excretory ducts open within the boundary of the oral cavity; consequently we will have to consider the mixed secretions of the parotid, the sub-maxillary, sublingual and the mucous glands. These latter are named "labial," "buccal," and "lingual," owing to their location. It is this, the common or mixed saliva, that interests us as dentists in a practical point of view. To the physiologist or physiological chemist, a thorough and minute knowledge of the chemical properties, including a correct analysis of the secretions of each individual salivary gland, is not only interesting but quite imperative; but from our stand-point of observation we care more for the effects produced—if any—by the oral secretions upon the teeth, and as these are brought about by the agency of the mixed saliva, we will proceed to consider it as such.

Probably no secretion of the human body has given chemists more trouble to analyze, and none has given more varying results, than the saliva.

Even the same saliva, analyzed by the same person will frequently differ quite materially in the quantity and quality of its constituents. In making an analysis of the saliva, the first question that naturally presents itself is this: "Will it give an acid or alkaline reaction?" In two hundred examinations made by an eminent member of our profession, who is here with us to-day, upon a like number of apparently healthy individuals, he found the saliva acid in one hundred and ninety-eight cases; two cases in which it was neutral.

In most of these cases a slightly alkaline reaction was observed, however, just before eating; but as soon as the meal was over, its character was immediately changed to an acid fluid, and remained so until the time for eating arrived again. Of about fifty mouths examined by myself, two or three were found neutral and the rest acid. These latter examinations were made from one to three hours after eating. It is held that acidity of the saliva is an indication of disease; that it is abnormal. Still we find this condition present in the mouths of people who have the appearance of perfect health and teeth entirely free from decay. The fact is, most of us are living artificial lives in our dress, habits of body, and the food we eat. If we were living in a perfectly physiological state; if we took our food as furnished us by nature, before it had passed through the hands of men to be deprived of its life-sustaining elements and tissue-making principles, the results might be different; for no one will question the fact that the character of all the secretions of the body, the saliva included, depends to a great extent upon our manner of living.

By extrinsic examinations made upon animals by the experimenter heretofore referred to, he found that animals which were free to roam wherever they chose in search of food and drink, that almost invariably their saliva gave an alkaline reaction; while on the other hand, stall-fed animals, animals that were not allowed sufficient exercise, that were deprived of the beneficial influence of the sun, that were fed upon slop and kept in damp, hot stables, and were living an entirely artificial life, that the saliva of these animals gave a decidedly acid reaction.

In alkaline saliva the analysis shows the alkalinity to be due to the presence of tri-sodium phosphate. The acidity may be due to any of the following acids, viz: Acetic, lactic, oxalic, uric, hydrochloric, nitric, and sulphuric.

The three first are formed probably and become constituents of the saliva, either in the secreting gland itself, or in the mouth by the decomposition of mucus, epithelium, or food remaining about the teeth. These acids, with hydrochloric have been found in the saliva by actual analysis. The latter —hydrochloric—is probably formed in most cases by galvanic action, which decomposes the chlorides and water, thus furnishing the necessary elements for its composition ; and particularly is this the case when there are different oxidizable metals in the mouth, thus presenting all the apparatus for a complete battery.

The presence of uric acid in the mouth may be caused by retention of the urine, or by disease of the kidneys, which fail to eliminate it from the blood. Nitric and sulphuric acid have never been detected, as far as my knowledge extends, in the saliva, but nevertheless they are thought by some to be present in that fluid under certain circumstances, from the effects which are observed upon the teeth.

You probably have all seen mouths in such a condition that it would not have excited the least surprise in your minds if told there existed any chemical combination whatever—mouths filthy beyond comparison. In such mouths I not only believe the conditions are present for the formation of the two last named acids, but I believe they are formed and act chemically upon the teeth.

We know that sulphur is present in albumen and other organic compounds that exist as proximate principles of a large class of food. Now, during the decomposition of any of these substances, sulphur is eliminated, and being in a nascent state will combine either with oxygen, forming sulphur*ous* acid, or with hydrogen, forming sulphureted hydrogen, and either of these might be so acted upon by other elements as to result in the formation of sulphu*ric* acid.

Nitrogen also being a constituent of a large class of aliment, is eliminated by the decomposition of the compound, either in company with oxygen, with which it forms five different compounds, or with hydrogen, forming the well-known compound ammonia. Either of the above combinations of nitrogen are capable of further change, and may finally terminate in nitric acid.

The most reliable analysis of saliva we have, gives in 1000 parts about 990 of water and 10 of solid material. In the latter are included the organic principle ptyalin, chlorides of potassium and sodium, sulphocyanide of potassium, phosphates and carbonates, epithelium ; and in alkaline saliva, tri-sodium phosphate.

The presence of sulphocyanide of potassium is proven by the blood-red tint imparted to saliva by the addition of the sesquichloride of iron. If the sulphocyanide of iron is formed, by the application of heat the color will temporarily disappear ; or by the addition of bi-chloride of mercury the color is entirely destroyed.

Mixed saliva possesses other chemical properties besides alkalinity and acidity ; it has the extraordinary metamorphic power of converting starch into sugar with wonderful rapidity. Numerous experiments have been made with the view of ascertaining what particular component so acted upon starch as to produce this interesting phenomenon, and all go to show that it is due to the organic principle ptyalin, together with the mucus of the mouth.

Enough has been said now to prove that the saliva exerts a great influence upon the teeth, depending upon its chemical conditions. It may cause and promote decay, or it may effectually prevent it, as the case may be. Want of time prevents us from reviewing the physiological and interesting properties of the saliva, and I will hasten to consider the one other great action it has upon the teeth, second only in importance to the part it plays in dental caries. I refer to the deposition from it of the elements composing salivary calculus, or tartar.

But few mouths, if undisturbed by either brush or pick, would be found exempt from this deposit ; while in the large majority of those that receive the ordinary attention of a most thorough washing, at least once a day, will be found traces, at least of tartar. The normal proportion of phosphate of lime is about 6 parts in every 1000 of saliva. This proportion may be morbidly increased, of course, as Dr.

Wright found in one case 14 parts in 1000. That tartar is formed by the precipitation of this phosphate of lime and other insoluble substances from the saliva, there cannot be a doubt.

I will now quote from an article bearing directly upon this subject, written by myself and published in the *Missouri Dental Journal*, Vol. IV., No: 5.

" We find that the most reliable analysis of tartar gives from 60 to 75 per cent. of strictly inorganic matter, the balance being made up of mucus, fibrin, epithelium and water. The inorganic portion is that with which we have to deal.

We have seen that the phosphate of lime exists as phosphate of lime in the saliva; hence it is simply precipitated from this fluid, undergoing no chemical change.

The saliva from all the glands, while being secreted and passing from the mouths of the ducts, is normal, but after it has once become mixed, or even as soon as it becomes exposed to the air, it is immediately changed from an alkaline to a neutral or slightly acid fluid. This can easily be proven by any one with litmus paper. This change may be brought about in two ways: First. The acids resulting from the putrefactive decomposition of particles of food or epithelium (the presence of which is the chief if not the only cause of dental caries) coming in contact with the saliva, would instantly unite with the free soda, and, if powerful enough, decompose the lactates, setting free lactic acid and uniting with the bases, forming salts ; thus, if hydrochloric acid were present, there would be formed the chlorides of calcium, sodium and potassium, with the liberation of lactic acid.

Second. The neutrality or acidity might be brought about by the carbonic acid, which passes off from the system in large quantities by each exhalation of air from the lungs. This coming in contact with every portion of the saliva as soon as it is emptied into the mouth, would tend to transform it immediately to an acid fluid ; and particularly would this be the case in the mouth of one who exhaled through this cavity instead of the nostrils.

The saliva now being changed chemically, the conditions favorable to the solution of the phosphate of lime, of course, are now present, and this substance is precipitated as an insoluble powder. It now finds its way between the margins of the gums and necks of the teeth, and is slowly but constantly deposited, first in the interstices of the enamel, and thus gets a firm hold, and then layer upon layer is piled on, until, in time, if nothing interferes, there is formed a huge mass, which becomes the seat of disease and corruption, and even of animal life. Now, from whence is the carbonate of lime? We have already seen that the mixed saliva is slightly impregnated with carbonic acid coming from the lungs; this, then, might act upon the lactates of lime, soda and potassa, forming carbonates of them all, and we would have carbonate of soda and carbonate of potassa. Now it is a well known chemical law, or fact, that carbonates of the alkali metals, such as potassium, sodium, ammonium, are soluble, while the carbonates of the alkaline earths, such as calcium, strontium and barium, are insoluble. The carbonate of lime belonging to this latter class, and therefore insoluble, would be precipitated as a white powder, and, like the phosphate of lime, would lodge in all protected places on the teeth, and with it make up the bulk of tartar as found in the human mouth. During sleep the conditions are most favorable for its rapid formation; it is then that the parts are in almost perfect rest; hence it remains when deposited. Now it is that respiration proceeds through the mouth, and the supply of carbonic acid is abundant.

From the foregoing will be seen the importance of preserving the normal alkalinity of the saliva, for upon this property depends its power in retaining in solution the principal ingredient of tartar or salivary calculus.

